# Hashimoto’s thyroiditis is associated with reduced invasiveness in papillary thyroid carcinoma: a propensity score-matched retrospective cohort study

**DOI:** 10.3389/fimmu.2025.1745452

**Published:** 2026-01-21

**Authors:** Rui Li, Xu Sun, Liai Hu, Zhiyuan Yu, Na Liu, Peiyu Li, Xudong Zhao, Wenquan Liang

**Affiliations:** 1 Medical School of Chinese PLA, Beijing, China; 2 Department of General Surgery, The First Medical Center, Chinese PLA General Hospital., Beijing, China; 3 School of Medicine, Nankai University, Tianjin, China; 4 Department of General Surgery, Beijing Friendship Hospital, Capital Medical University, Beijing, China

**Keywords:** Hashimoto’s thyroiditis, papillary thyroid cancer, predictors, risk factors, thyroid cancer

## Abstract

**Background:**

Papillary thyroid cancer (PTC), the predominant histologic subtype of thyroid cancer cases, has increased substantially over the past decades. In previous studies, Hashimoto’s thyroiditis (HT) exerts a paradoxical dual role in PTC. However, limited studies have specifically examined the association between HT and the invasion of PTC.

**Methods:**

In this retrospective study, 10329 PTC patients were selected, and the clinicopathological features were retrospectively analyzed. Propensity score matching (PSM) was employed to minimize confounding effects from baseline variables. Univariate analysis and multivariate analysis were performed using binary logistic regression to determine the predictive factor. Odds ratio (OR) and 95% confidence interval (CI) were calculated.

**Results:**

Among 10329 PTC patients, 992 (9.6%) individuals were diagnosed with HT. Compared to the non-HT group, the HT group demonstrated lower rates of extrathyroidal extension (p<0.001), reduced multifocality (p=0.004), decreased bilateral carcinoma involvement (p<0.001), and a greater proportion of pathological N0 stage tumors (p=0.003). Following PSM, a cohort of 970 patients with HT and 2783 non-HT controls without HT were analyzed. HT was significantly associated with lower rates of: central lymph node metastasis (CLNM) in cN0 patients (p<0.001); lateral lymph node metastasis (LLNM) in cN1b patients (p=0.008); and bilateral carcinoma detection in patients with clinically unilateral PTC lesions (p=0.001).

**Conclusion:**

This study found an association between HT and reduced invasiveness of PTC, as evidenced by increased node-negative disease and reduced bilateral pathological involvement.

## Introduction

The incidence of thyroid cancer (TC) has increased substantially over the past decades, ranking as the seventh most prevalent cancer globally and the fifth most prevalent malignancy in females (1–3). Papillary thyroid cancer (PTC), the predominant histologic subtype of TC cases, represents the primary driver of this observed epidemiological rise (4). Updated in 2025, the American Thyroid Association (ATA) Management Guidelines for Adult Patients with Thyroid Nodules and Differentiated Thyroid Cancer further mandate precise risk stratification and personalized treatment planning for PTC patients (5).

Hashimoto’s thyroiditis (HT), one of the most prevalent autoimmune thyroid disorders, arises from complex interactions among genetic susceptibility, environmental triggers, and epigenetic modifications (6). It is serologically characterized by elevated anti-thyroglobulin (TgAb) and anti-thyroid peroxidase (TPOAb) antibodies. Histopathologically, HT manifests as lymphoplasmacytic infiltration, germinal center formation within lymphoid follicles, and progressive parenchymal atrophy (7). The relationship between HT and PTC remains contentious. On the one hand, chronic inflammation-driven oncogenic transformation and TSH-mediated follicular epithelial proliferation may promote PTC development (8, 9). Conversely, Anil et al. prospectively demonstrated no increased risk in HT patients with thyroid nodules (10). However, few studies have examined the association between HT and PTC invasion.

In this study, we systematically evaluated the association between clinicopathological characteristics and HT status in PTC patients. Propensity score matching (PSM) was employed to minimize confounding effects from baseline variables. Primary endpoints included lymph node metastasis status and incidence of bilateral carcinomas (initially diagnosed as unilateral disease preoperatively). Our comprehensive analysis provides clinically actionable evidence for refining risk-adapted management strategies in HT-associated PTC.

## Materials and methods

### Patients’ selection

Consecutive PTC patients who underwent surgical resection for treatment at the First Medical Center of the Chinese People’s Liberation Army (PLA) General Hospital from January 2020 to February 2025 were screened. Related patients meeting the following criteria were selected: (1) an obvious pathologic diagnosis of PTC; (2) primary cases without a history of thyroid tumors; (3) underwent surgical resection. The exclusion criteria were as follows: (1) patients with a history of thyroid tumors; (2) patients with other types of thyroid tumors, including follicular thyroid cancer, medullary thyroid cancer, and anaplastic thyroid cancer; (3) patients with evidence of distant metastatic disease; (4) patients who received ablation of thyroid nodules. This study was conducted in accordance with the Declaration of Helsinki and approved by the Medical Ethics Committee of the First Medical Center of the Chinese PLA General Hospital.

### Data collection

The preoperative and postoperative clinicopathological features were retrospectively studied. Baseline variables, such as patient age and gender, were obtained from medical records. The clinical N stage and tumor location were determined from preoperative ultrasound and computed tomography scans. The surgical strategy for PTC, including thyroid lobectomy/total thyroidectomy, central compartment neck dissection, and lateral neck dissection (for cN1b patients), was determined by the surgeon based on preoperative staging. The presence of HT was diagnosed postoperatively by histopathological examination of paraffin-embedded sections, characterized by diffuse lymphoplasmacytic infiltration, and the presence of lymphoid follicles with reactive germinal centers in the background thyroid tissue, irrespective of preoperative antibody status (11). Tumor size, extrathyroidal extension, multifocal carcinoma, bilateral carcinoma, central lymph node metastasis (CLNM) count, central lymph node dissection (CLND) yield, pathological N stage (pN), lateral lymph node metastasis (LLNM) count, and lateral lymph node dissection (LLND) yield were determined through postoperative histopathological examination.

### Statistical analysis

IBM SPSS Statistics 26.0 software (SPSS Inc, Chicago, IL, USA) and R software (ver. 4.2.1, R Development Core Team) were adopted for statistical analyses in this study. Numerical data with normal distribution were expressed as the mean ± standard deviation (SD), and numerical data without normal distribution were expressed as median (interquartile range, IQR). Categorical data are presented as absolute numbers and percentages. The MatchIt package was used to conduct the PSM, with a matching ratio of 1:3 between the HT and non-HT groups and a matching capacity of 0.02 (12). The standardized mean differences (SMD) of covariate balance before and after PSM were subsequently calculated. Univariate and multivariate analyses were performed using binary logistic regression to identify the predictive factors for CLNM, LLNM, and bilateral cancer. Odds ratio (OR) and 95% confidence interval (CI) were calculated. *P*-values were two-tailed, and *P* < 0.05 was considered statistically significant. Data visualization was performed using GraphPad Prism 8 Software.

## Results

### Patient selection and characteristics

**Figure 1:**
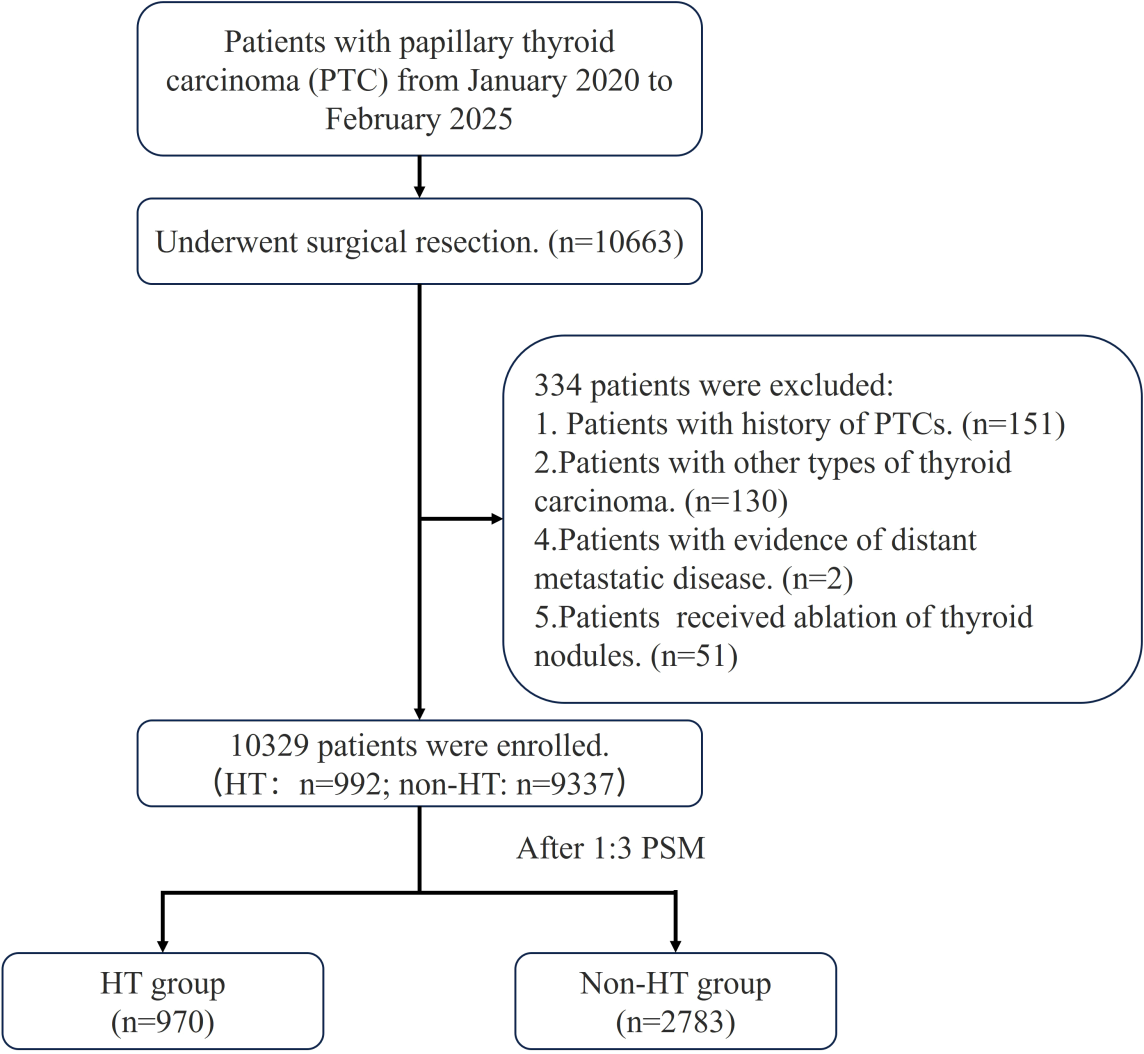
The flow chart for patient selection PTC, papillary thyroid carcinoma; HT, Hashimoto’s thyroiditis; PSM, propensity score matching.

**Figure 2:**
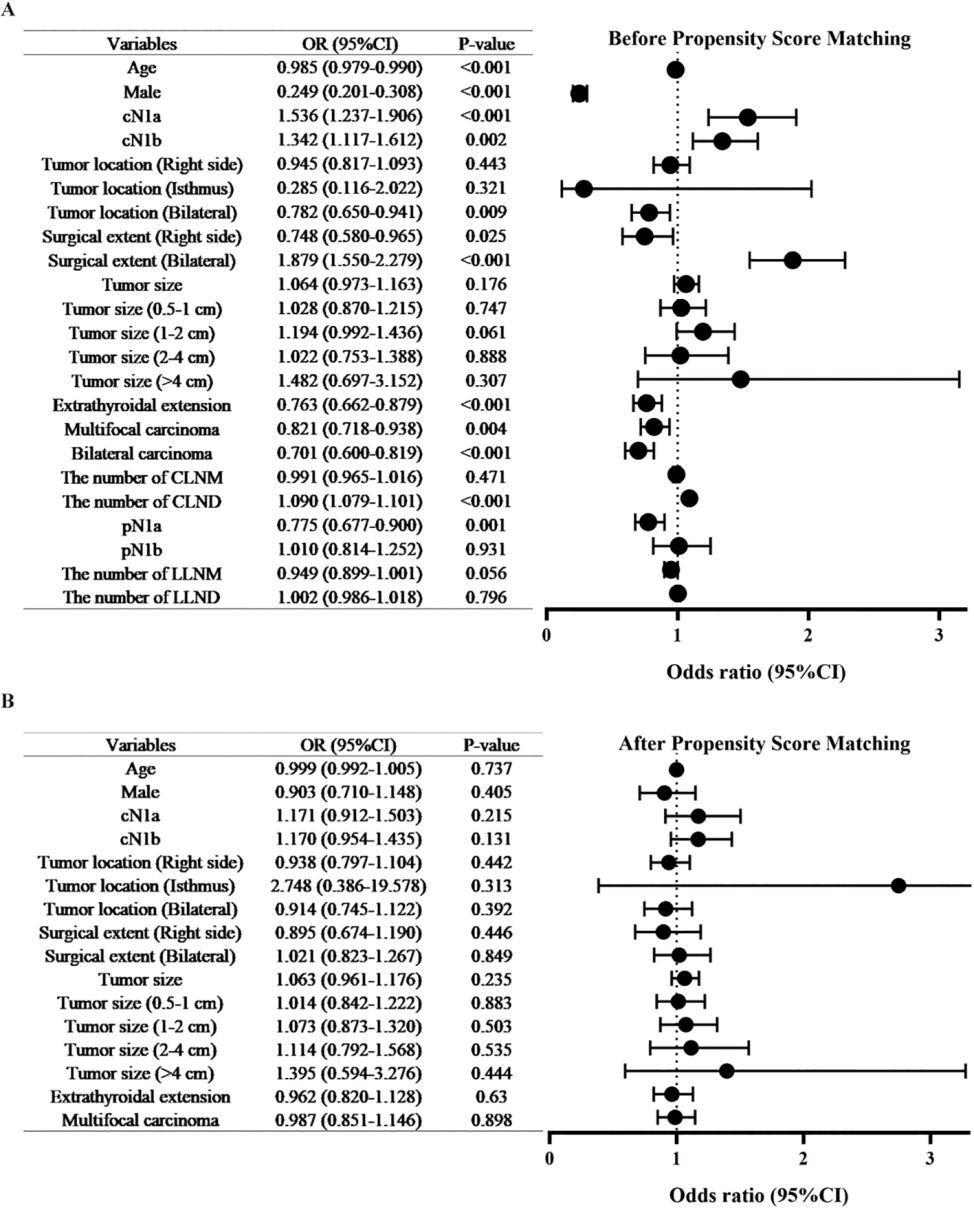
Comparison of clinical characteristics in papillary thyroid carcinoma patients with versus without Hashimoto’s thyroiditis before and after propensity score matching. **(A)** Pre-matching cohort; **(B)** Post-matching cohort. CLNM, central lymph node metastasis, CLND, central lymph node dissection, LLNM, lateral lymph node metastasis, LLND, lateral lymph node dissection.

Initially, a cohort of 10663 PTC patients who underwent thyroidectomy was included. Of these, 151 patients had a history of PTCs, 130 patients had other types of thyroid carcinoma, 2 patients had evidence of distant metastatic disease, and 51 patients had received ablation of thyroid nodules. After rigorous screening, 10329 patients were selected for further analysis, and 992 (9.6%) were diagnosed with HT (**Figure 1**). Compared with the non-HT group, the HT group exhibited a significantly younger age (p<0.001), greater female predominance (p<0.001), higher rates of clinical N1a and N1b disease (p<0.001), and more frequent bilateral thyroidectomy (p<0.001). Conversely, the HT group demonstrated lower rates of extrathyroidal extension (p<0.001), reduced multifocality (p=0.004), and decreased bilateral carcinoma involvement (p<0.001). Additionally, the HT group had higher numbers of CLND (p<0.001) and a greater proportion of pathological N0 stage tumors (p=0.003) (**Table 1**; **Figure 2A**).

**Table 1:** Clinical characteristics of PTC patients before propensity score matching.

Characteristic	Patients (N = 10329)			*P* value	OR (95%CI)
	Total (n=10329)	HT absent (n = 9337)	HT present (n = 992)		
Age ^a^, years	43 (35-52)	44 (35-52)	41.5 (33-51)	<0.001	0.985 (0.979-0.990)
Gender, n (%)				<0.001	0.249 (0.201-0.308)
Female	7376 (71.4)	6482 (69.4)	894 (90.1)		
Male	2953 (28.6)	2855 (30.6)	98 (9.9)		
Clinical N stage, n (%)				<0.001	
cN0	8160 (79.0)	7432 (79.6)	728 (73.4)		
cN1a	826 (8.0)	718 (7.7)	108 (10.9)	<0.001	1.536 (1.237-1.906)
cN1b	1343 (13.0)	1187 (12.7)	156 (15.7)	0.002	1.342 (1.117-1.612)
Clinical tumor location, n (%)				0.051	
Left side	3746 (36.3)	3361 (36.0)	385 (38.8)		
Right side	4332 (41.9)	3909 (41.9)	423 (42.6)	0.443	0.945 (0.817-1.093)
Isthmus	38 (0.4)	36 (0.4)	2 (0.2)	0.321	0.285 (0.116-2.022)
Bilateral	2213 (21.4)	2031 (21.8)	182 (18.3)	0.009	0.782 (0.650-0.941)
Surgical extent, n (%)				<0.001	
Left side	1926 (18.6)	1794 (19.2)	132 (13.3)		
Right side	2320 (22.5)	2199 (23.6)	121 (12.2)	0.025	0.748 (0.580-0.965)
Bilateral	6083 (58.9)	5344 (57.2)	739 (74.5)	<0.001	1.879 (1.550-2.279)
Tumor size ^a^, cm	0.8 (0.6-1.2)	0.8 (0.5-1.2)	0.8 (0.6-1.2)	0.176	1.064 (0.973-1.163)
Tumor size, stratified, n (%)				0.259	
<0.5 cm	2575 (24.9)	2341 (25.1)	234 (23.6)		
0.5-1 cm	2583 (44.4)	4156 (44.5)	427 (43.0)	0.747	1.028 (0.870-1.215)
1-2 cm	2505 (24.3)	2238 (24.0)	267 (26.9)	0.061	1.194 (0.992-1.436)
2-4 cm	604 (5.8)	548 (5.9)	56 (5.6)	0.888	1.022 (0.753-1.388)
>4 cm	62 (0.6)	54 (0.6)	8 (0.8)	0.307	1.482 (0.697-3.152)
Extrathyroidal extension, n (%)				<0.001	0.763 (0.662-0.879)
Absence	2732 (26.4)	2420 (25.9)	312 (31.5)		
Presence	7597 (73.6)	6917 (74.1)	680 (68.5)		
Multifocal carcinoma, n (%)				0.004	0.821 (0.718-0.938)
Absence	5777 (55.9)	5179 (55.5)	598 (60.3)		
Presence	4552 (44.1)	4158 (44.5)	394 (39.7)		
Bilateral carcinoma, n (%)				<0.001	0.701 (0.600-0.819)
Absence	7386 (71.5)	6616 (70.9)	770 (77.6)		
Presence	2943 (28.5)	2721 (29.1)	222 (22.4)		
The number of CLNM ^a^	0 (0-2)	0 (0-2)	0 (0-2)	0.471	0.991 (0.965-1.016)
The number of CLND ^a^	4 (2-8)	4 (1-7)	7 (3-12)	<0.001	1.090 (1.079-1.101)
Pathological N stage, n (%)				0.003	
N0	5942 (57.5)	5331 (57.1)	611 (61.6)		
N1a	3336 (32.3)	3064 (32.8)	272 (27.4)	0.001	0.775 (0.677-0.900)
N1b	1051 (10.2)	942 (10.1)	109 (11.0)	0.931	1.010 (0.814-1.252)
Patients with LLND, n=1343					
The number of LLNM ^a^	2 (1-5)	2 (1-5)	2 (0-4)	0.056	0.949 (0.899-1.001)
The number of LLND ^a^	14 (9-20)	13 (9-20)	14 (10-21)	0.796	1.002 (0.986-1.018)

a Values are presented as median (Q1-Q3).

PTC, papillary thyroid carcinoma; HT, Hashimoto's thyroiditis; CLNM, central lymph node metastasis; CLND, central lymph node dissection; LLNM, lateral lymph node metastasis; LLND, lateral lymph node dissection.

**Figure 3:**
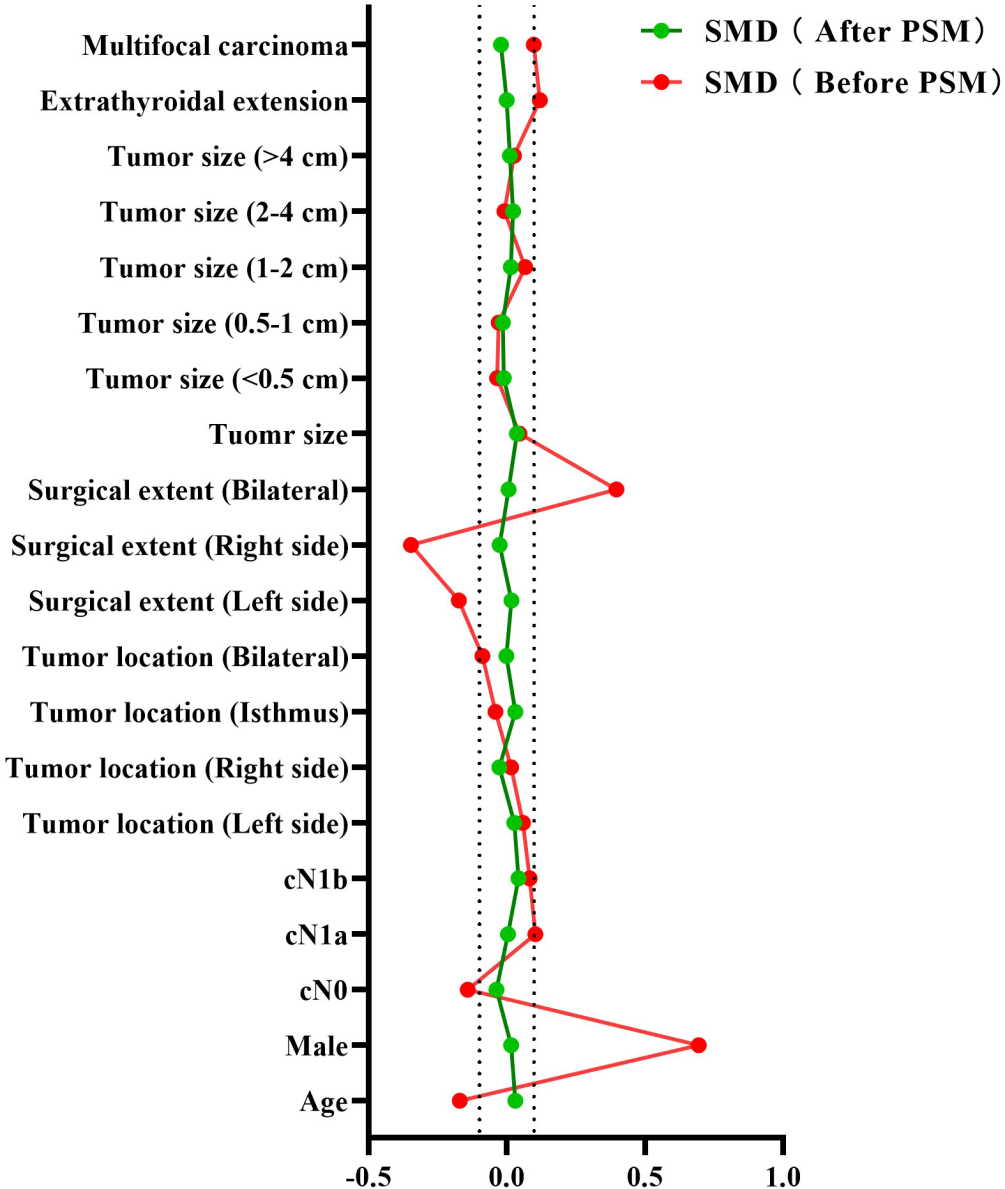
The assessment of standardized mean differences (SMD) for covariate balance before and after propensity score matching (PSM).

Covariates, including age, gender, clinical N stage, clinical tumor location, surgical extent, tumor size, extrathyroidal extension, and multifocal carcinoma, were well-balanced through PSM. Following a 1:3 ratio PSM, the final cohort comprised 970 patients with HT and 2783 non-HT controls. All covariates demonstrated non-significant intergroup differences (*P* > 0.05; **Figure 2B**; **Supplementary Table 1**) with SMD below the 0.1 threshold (**Table 2**; **Figure 3**). The propensity score distribution and matching methodology are detailed in **Supplementary Figures 2, 3**.

**Table 2:** Clinical characteristics of PTC patients after 1:3 propensity score matching.

Characteristic	Patients (N = 3753)			*P* value	SMD	
	Total (n=3753)	HT absent (n = 2783)	HT present (n = 970)		Before PSM	After PSM
Age ^a^, years	42 (34-50)	42 (34-50)	42 (33-51)	0.737	-0.170	0.031
Gender, n (%)				0.405		
Female	3347 (89.2)	2475 (88.9)	872 (89.9)		0.694	0.016
Male	406 (10.8)	308 (11.1)	98 (10.1)		-0.694	-0.016
Clinical N stage, n (%)				0.187		
cN0	2859 (76.2)	2141 (76.9)	718 (74.0)		-0.141	-0.038
cN1a	344 (9.2)	247 (8.9)	97 (10.0)	0.215	0.103	0.004
cN1b	550 (14.7)	395 (14.2)	155 (16.0)	0.131	0.083	0.042
Clinical tumor location, n (%)				0.540		
Left side	1413 (37.6)	1036 (37.2)	377 (38.9)		0.058	0.027
Right side	1607 (42.8)	1198 (43.0)	409 (42.2)	0.442	0.016	-0.027
Isthmus	4 (0.1)	2 (0.1)	2 (0.2)	0.313	-0.041	0.031
Bilateral	729 (19.4)	547 (19.7)	182 (18.8)	0.392	-0.088	-0.002
Surgical extent, n (%)				0.507		
Left side	511 (13.6)	379 (13.6)	132 (13.6)		-0.174	0.017
Right side	509 (13.6)	388 (13.9)	121 (12.5)	0.446	-0.347	-0.026
Bilateral	2733 (72.8)	2016 (72.4)	717 (73.9)	0.849	0.397	0.006
Tumor size ^a^, cm	0.8 (0.6-1.2)	0.8 (0.6-1.2)	0.8 (0.6-1.2)	0.235	0.046	0.036
Tumor size, stratified, n (%)				0.863		
<0.5 cm	908 (24.2)	679 (24.4)	229 (23.6)		-0.035	-0.011
0.5-1 cm	1648 (43.9)	1228 (44.1)	420 (43.3)	0.883	-0.030	-0.015
1-2 cm	967 (25.8)	710 (25.5)	257 (26.5)	0.503	0.066	0.014
2-4 cm	205 (5.5)	149 (5.4)	56 (5.8)	0.535	-0.009	0.022
>4 cm	25 (0.7)	17 (0.6)	8 (0.8)	0.444	0.026	0.011
Extrathyroidal extension				0.630		
Absence	1107 (29.5)	815 (29.3)	292 (30.1)		0.119	-0.0003
Presence	2646 (70.5)	1968 (70.7)	678 (69.9)		-0.119	0.0003
Multifocal carcinoma				0.898		
Absence	1107 (29.5)	1647 (59.2)	577 (59.5)		0.098	-0.021
Presence	2646 (70.5)	1136 (40.8)	393 (40.5)		-0.098	0.021

a Values are presented as median (Q1-Q3).

PTC, papillary thyroid carcinoma; HT, Hashimoto's thyroiditis; SMD, standardized mean differences.

### HT independently associates with lower CLNM in cN0 PTC

**Figure 4:**
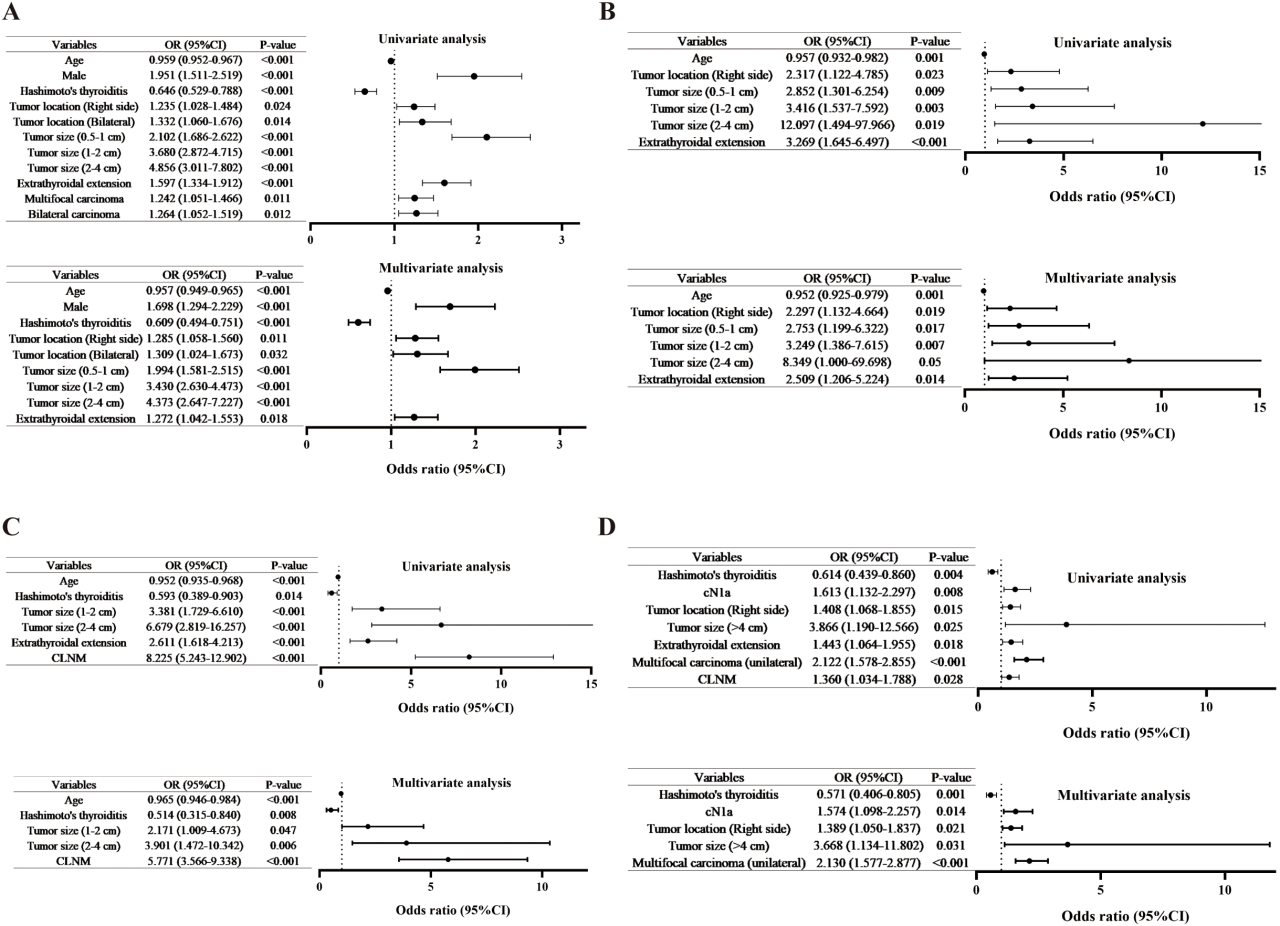
Univariate and multivariate logistic regression analyses of risk factors for: **(A)** CLNM in cN0 PTC patients; **(B)** CLNM in cN1a PTC patients; **(C)** LLNM in cN1b PTC patients; **(D)** bilateral carcinoma identified in clinically unilateral PTC lesions. CLNM, central lymph node metastasis, LLNM, lateral lymph node metastasis, PTC, papillary thyroid cancer.

After propensity score matching, 3753 patients were included in the final analysis. Among them, 2859 patients (76.2%) were clinically node-negative (cN0), of whom 809 (28.3%) had pathologically confirmed CLNM. Univariate analysis identified male gender (p<0.001; OR = 1.951), right-sided tumors (p=0.024; OR = 1.235), bilateral tumors (p=0.014; OR = 1.332), larger tumor size (p < 0.001), extrathyroidal extension (p<0.001; OR = 1.597), multifocal carcinoma (p=0.011; OR = 1.242), and bilateral carcinoma (p=0.012; OR = 1.264) as potential risk factors for CLNM. In contrast, increasing age (p<0.001; OR = 0.959) and HT (p < 0.001; OR = 0.646) showed potential protective effects. Furthermore, a multivariate analysis was conducted using binary logistic regression to identify the predictive factors for CLNM in cN0 PTC patients (**Table 3**; **Figure 4A**). Male gender (p<0.001; OR = 1.698), right-sided tumors (p=0.011; OR = 1.285), bilateral tumors (p=0.032; OR = 1.309), larger tumor size (p<0.001), and extrathyroidal extension (p=0.018; OR = 1.272) as significant risk factors for CLNM, while increasing age (p<0.001; OR = 0.957) and HT (p<0.001; OR = 0.609) were significant associated with lower CLNM rate in cN0 PTC.

**Table 3:** The risk factors for central cervical lymph node metastasis in cN0 PTC patients (N=2859).

	pLN (-) N=2050 (%)	pLN (+) N=809 (%)	Univariate analysis		Multivariate analysis	
			*P*-value	OR (95%CI)	*P*-value	OR (95%CI)
Age ^a^, years	45 (36-53)	38 (32-48)	<0.001	0.959 (0.952-0.967)	<0.001	0.957 (0.949-0.965)
Gender, n (%)			<0.001	1.951 (1.511-2.519)	<0.001	1.698 (1.294-2.229)
Female	1891 (92.2)	695 (85.9)				
Male	159 (7.8)	114 (14.1)				
HT, n (%)			<0.001	0.646 (0.529-0.788)	<0.001	0.609 (0.494-0.751)
Absence	1490 (72.7)	651 (80.5)				
Presence	560 (27.3)	158 (19.5)				
Clinical tumor location, n (%)			0.037		0.05	
Left side	802 (39.1)	273 (33.7)				
Right side	880 (42.9)	370 (45.7)	0.024	1.235 (1.028-1.484)	0.011	1.285 (1.058-1.560)
Isthmus	2 (0.1)	0 (0)	0.999		0.999	
Bilateral	366 (17.9)	166 (20.5)	0.014	1.332 (1.060-1.676)	0.032	1.309 (1.024-1.673)
Surgical extent, n (%)			0.091			
Left side	357 (17.4)	119 (14.7)				
Right side	335 (16.3)	153 (18.9)				
Bilateral	1358 (66.2)	537 (66.4)				
Tumor size ^a^,cm, n (%)			<0.001		<0.001	
<0.5 cm	685 (33.4)	131 (16.2)				
0.5-1 cm	965 (47.1)	388 (48.0)	<0.001	2.102 (1.686-2.622)	<0.001	1.994 (1.581-2.515)
1-2 cm	351 (17.1)	247 (30.5)	<0.001	3.680 (2.872-4.715)	<0.001	3.430 (2.630-4.473)
2-4 cm	42 (2.0)	39 (4.8)	<0.001	4.856 (3.011-7.802)	<0.001	4.373 (2.647-7.227)
>4 cm	7 (0.3)	4 (0.5)	0.084		0.155	
Extrathyroidal extension, n (%)			<0.001	1.597 (1.334-1.912)	0.018	1.272 (1.042-1.553)
Absence	751 (36.6)	215 (26.6)				
Presence	1299 (63.4)	594 (73.4)				
Multifocal carcinoma, n (%)			0.011	1.242 (1.051-1.466)	0.260	
Absence	1304 (63.6)	473 (58.5)				
Presence	746 (36.4)	336 (41.5)				
Bilateral carcinoma, n (%)			0.012	1.264 (1.052-1.519)	0.307	
Absence	1562 (76.2)	580 (71.7)				
Presence	488 (23.8)	229 (28.3)				

a Values are presented as median (Q1-Q3).

PTC, papillary thyroid carcinoma; HT, Hashimoto's thyroiditis.

Among 344 cN1aPTC patients, 291 (84.6%) had pathologically confirmed CLNM. After univariate and multivariate analysis, right-sided tumors (p=0.019; OR = 2.297), larger tumor size (p=0.027), and extrathyroidal extension (p=0.014; OR = 2.509) were identified as significant risk factors for CLNM, while increasing age (p=0.001; OR = 0.952) was a significant protective factor (**Table 4**; **Figure 4B**).

**Table 4:** The risk factors for central cervical lymph node metastasis in cN1a PTC patients (N=344).

	pLN (-) N=53 (%)	pLN (+) N=291 (%)	Univariate analysis		Multivariate analysis	
			*P*-value	OR (95%CI)	*P*-value	OR (95%CI)
Age ^a^, years	45 (34-55)	38 (32-46)	0.001	0.957 (0.932-0.982)	0.001	0.952 (0.925-0.979)
Gender, n (%)			0.152			
Female	47 (88.7)	234 (80.4)				
Male	6 (11.3)	57 (19.6)				
HT, n (%)			0.495			
Absence	36 (67.9)	211 (72.5)				
Presence	17 (32.1)	80 (27.5)				
Clinical tumor location, n (%)			0.058		0.128	
Left side	24 (45.3)	98 (33.7)				
Right side	13 (24.5)	123 (42.3)	0.023	2.317 (1.122-4.785)	0.019	2.297 (1.132-4.664)
Isthmus	1 (1.9)	1 (0.3)	0.326		0.900	
Bilateral	15 (28.3)	69 (23.7)	0.744		0.487	
Surgical extent, n (%)			0.188			
Left side	4 (7.5)	24 (8.2)				
Right side	0 (0)	17 (5.8)				
Bilateral	49 (92.5)	250 (85.9)				
Tumor size ^a^,cm, n (%)			0.006		0.027	
<0.5 cm	15 (28.3)	31 (10.7)				
0.5-1 cm	19 (35.8)	112 (38.5)	0.009	2.852 (1.301-6.254)	0.017	2.753 (1.199-6.322)
1-2 cm	17 (32.1)	120 (41.2)	0.003	3.416 (1.537-7.592)	0.007	3.249 (1.386-7.615)
2-4 cm	1 (1.9)	25 (8.6)	0.019	12.097 (1.494-97.966)	0.050	8.349 (1.000-69.698)
>4 cm	1 (1.9)	3 (1.0)	0.755		0.662	
Extrathyroidal extension, n (%)			<0.001	3.269 (1.645-6.497)	0.014	2.509 (1.206-5.224)
Absence	16 (30.2)	34 (11.7)				
Presence	37 (69.8)	257 (88.3)				
Multifocal carcinoma, n (%)			0.498			
Absence	28 (52.8)	139 (47.8)				
Presence	25 (47.2)	152 (52.2)				
Bilateral carcinoma, n (%)			0.481			
Absence	31 (58.5)	185 (63.6)				
Presence	22 (41.5)	106 (36.4)				

a Values are presented as median (Q1-Q3).

PTC, papillary thyroid carcinoma; HT, Hashimoto's thyroiditis.

### HT independently associates with lower LLNM in CN1b PTC

Of the 550 cN1b PTC patients studied, 422 (76.7%) developed pathologically proven LLNM, among whom 54 (12.8%) presented with skip metastasis (**Table 5**; **Figure 4C**). Univariate analysis revealed significant associations for LLNM risk: tumor size 1–2 cm (p<0.001, OR = 3.381); tumor size 2–4 cm (p<0.001, OR = 6.679); extrathyroidal extension (p<0.001, OR = 2.611); and CLNM (p<0.001, OR = 8.225). Protective effects were observed for age (p<0.001, OR = 0.952) and HT (p=0.014, OR = 0.593). Multivariate analysis confirmed independent predictors: tumor size 1–2 cm (p=0.047, OR = 2.171); tumor size 2–4 cm (p=0.006, OR = 3.901); CLNM (p<0.001, OR = 5.771); with persistent protection from age (p<0.001, OR = 0.965).HT (p=0.008, OR = 0.514) was significantly associated with lower LLNM rate in cN1b PTC.

**Table 5:** The risk factors for lateral cervical lymph node metastasis in cN1b PTC patients (N=550).

	pLN (-) N=128 (%)	pLN (+) N=422 (%)	Univariate analysis		Multivariate analysis	
			*P*-value	OR (95%CI)	*P*-value	OR (95%CI)
Age ^a^, years	44 (34-52)	36 (30-43)	<0.001	0.952 (0.935-0.968)	<0.001	0.965 (0.946-0.984)
Gender, n (%)			0.488			
Female	114 (89.1)	366 (86.7)				
Male	14 (10.9)	56 (13.3)				
HT, n (%)			0.014	0.593 (0.389-0.903)	0.008	0.514 (0.315-0.840)
Absence	81 (63.3)	314 (74.4)				
Presence	47 (36.7)	108 (25.6)				
Clinical tumor location, n (%)			0.228			
Left side	58 (45.3)	158 (37.4)				
Right side	44 (34.4)	177 (41.9)				
Isthmus	0 (0)	0 (0)				
Bilateral	26 (20.3)	87 (20.6)				
Tumor size ^a^,cm, n (%)			<0.001		0.004	
<0.5 cm	20 (15.6)	26 (6.2)				
0.5-1 cm	55 (43.0)	109 (25.8)	0.215		0.855	
1-2 cm	43 (33.6)	189 (44.8)	<0.001	3.381 (1.729-6.610)	0.047	2.171 (1.009-4.673)
2-4 cm	10 (7.8)	88 (20.9)	<0.001	6.679 (2.819-16.257)	0.006	3.901 (1.472-10.342)
>4 cm	0 (0)	10 (2.4)	NA		NA	
Extrathyroidal extension, n (%)			<0.001	2.611 (1.618-4.213)	0.427	
Absence	36 (28.1)	55 (13.0)				
Presence	92 (71.9)	367 (87.0)				
Multifocal carcinoma, n (%)			0.074			
Absence	74 (57.8)	206 (48.8)				
Presence	54 (42.2)	216 (51.2)				
Bilateral carcinoma, n (%)			0.970			
Absence	85 (66.4)	281 (66.6)				
Presence	43 (33.6)	141 (33.4)				
CLNM, n (%)			<0.001	8.225 (5.243-12.902)	<0.001	5.771 (3.566-9.338)
Absence	70 (54.7)	54 (12.8)				
Presence	58 (45.3)	368 (87.2)				

a Values are presented as median (Q1-Q3).

PTC, papillary thyroid carcinoma; HT, Hashimoto's thyroiditis; CLNM, central lymph node metastasis.

### HT independently associates with reduced bilateral pathological involvement

In our cohort, 1574 patients had clinically unilateral lesions and underwent bilateral thyroidectomy, involving 1361 cN0 patients and 213 cN1a patients. Among them, 256 patients (16.2%) were pathologically confirmed to have bilateral thyroid carcinoma (**Table 6**; **Figure 4D**). Univariate analysis identified cN1a metastasis (p=0.008, OR = 1.613), right-sided tumors (p=0.015, OR = 1.408), tumor size >4cm (p=0.025, OR = 3.866), extrathyroidal extension (p=0.018, OR = 1.443), unilateral multifocal carcinoma (p<0.001, OR = 2.122), and CLNM (p=0.028, OR = 1.360) as potential risk factors for bilateral carcinoma, while HT showed a protective association (p=0.004, OR = 0.614). Multivariate analysis confirmed cN1a (p=0.014, OR = 1.574), right-sided location (p=0.021, OR = 1.389), tumor size >4cm (p=0.031, OR = 3.668), and unilateral multifocal carcinoma (p<0.001, OR = 2.130) as independent risk factors, with HT remaining a significant negative correlate (p=0.001, OR = 0.571).

**Table 6:** The risk factors for bilateral carcinoma in clinical unilateral lesion PTC patients (N=1574).

	Unilateral N=1318 (%)	Bilateral N=256 (%)	Univariate analysis		Multivariate analysis	
			*P*-value	OR (95%CI)	*P*-value	OR (95%CI)
Age ^a^, years	45 (36-52)	43 (36-53)	0.727			
Gender, n (%)			0.913			
Female	1182 (89.7)	229 (89.5)				
Male	136 (10.3)	27 (10.5)				
HT, n (%)			0.004	0.614 (0.439-0.860)	0.001	0.571 (0.406-0.805)
Absence	958 (72.7)	208 (81.3)				
Presence	360 (27.3)	48 (18.7)				
Clinical N stage, n (%)			0.008	1.613 (1.132-2.297)	0.014	1.574 (1.098-2.257)
cN0	1153 (87.5)	208 (81.3)				
cN1a	165 (12.5)	48 (18.8)				
Clinical tumor location, n (%)			0.015	1.408 (1.068-1.855)	0.021	1.389 (1.050-1.837)
Left side	598 (45.4)	95 (37.1)				
Right side	720 (54.6)	161 (62.9)				
Tumor size ^a^,cm, n (%)			0.101		0.134	
<0.5 cm	341 (25.9)	63 (24.6)				
0.5-1 cm	587 (44.5)	103 (40.2)	0.767			
1-2 cm	323 (24.5)	69 (27.0)	0.447			
2-4 cm	60 (4.6)	16 (6.3)	0.241			
>4 cm	7 (0.5)	4 (2.0)	0.025	3.866 (1.190-12.566)	0.031	3.668 (1.134-11.802)
Extrathyroidal extension, n (%)			0.018	1.443 (1.064-1.955)	0.141	
Absence	434 (32.9)	65 (25.4)				
Presence	884 (67.1)	191 (74.6)				
Multifocal carcinoma (unilateral), n (%)			<0.001	2.122 (1.578-2.855)	<0.001	2.130 (1.577-2.877)
Absence	1075(81.6)	173 (67.6)				
Presence	243 (18.4)	83 (32.4)				
CLNM, n (%)			0.028	1.360 (1.034-1.788)	0.470	
Absence	872 (66.2)	151 (59.0)				
Presence	446 (33.8)	105 (41.0)				

a Values are presented as median (Q1-Q3).

PTC, papillary thyroid carcinoma; HT, Hashimoto's thyroiditis; CLNM, central lymph node metastasis.

## Discussion

The imperative for precision risk stratification in PTC management is substantial, which is complicated by concomitant HT. This complexity arises not only from HT-induced diagnostic pitfalls in fine-needle aspiration biopsy (FNAB), but more critically from the paradoxical oncologic implications of HT itself (13, 14). In our cohort, HT coexisted with PTC in 9.6% of cases, demonstrating significant female predominance, consistent with prior epidemiological reports (11, 15–17). Crucially, the presence of HT prompted more aggressive preoperative management, evidenced by significantly higher rates of clinically suspected nodal metastases (cN1a and cN1b stage) and consequent performance of bilateral thyroidectomy. However, postoperative pathological assessment revealed a paradoxical protective association: HT was independently associated with reduced tumor invasiveness, with significantly lower frequencies of extrathyroidal extension, multifocality, and bilateral carcinoma involvement, and a higher proportion of pN0 stage tumors. After PSM to control for key confounders, HT consistently demonstrated an association with reduced PTC invasiveness. This was evidenced by significantly reduced CLNM rates in cN0 patients, lower incidence of LLNM in cN1b cases, and decreased bilateral tumor detection among preoperatively diagnosed unilateral tumors. These robust findings establish a significant association between HT and diminished invasive potential of PTC.

The protective association between HT and attenuated PTC invasiveness observed in our PSM analysis aligns with robust existing evidence. A multicenter cohort of 9210 PTC patients demonstrated HT’s negative correlation with aggressive features, including primary tumors larger than 4 cm, gross extrathyroidal extension, extranodal extension, and distant metastasis, and concurrently correlated with superior 10-year disease-specific and recurrence-free survival (11). This is further substantiated by a meta-analysis of 10648 cases across 38 studies, which found that HT co-occurrence significantly predicted reduced extrathyroidal extension, fewer positive lymph nodes, and improved long-term recurrence-free survival (17). Single-institutional data from 435 surgically treated PTC patients corroborated these findings, showing that PTC-HT cases had smaller tumor dimensions, less lymph node involvement, and earlier-stage disease than HT-negative counterparts (18). Collectively, these findings reinforce HT’s role in contributing to its less aggressive clinicopathological presentation in PTC.

Notwithstanding the predominant protective patterns, certain studies report conflicting associations between Hashimoto’s thyroiditis and papillary thyroid carcinoma behavior. Lee et al.’s aforementioned meta-analysis paradoxically identified HT as a risk factor for multifocal tumor involvement (17), a finding corroborated by Cappellacci et al. in their cohort of 839 PTC patients, in which the HT group demonstrated significantly higher multifocal rates (40.5% vs. 29.6%) (19). Although the latter study confirmed HT’s association with smaller tumor diameters, their multivariate analysis unexpectedly established HT as an independent risk factor for thyroid cancer development. This aligns with Apostolou et al.’s investigation of 3233 patients with multinodular thyroid disease, in which HT independently predicted malignant histopathology (20). Most strikingly, Chen et al.’s cohort demonstrated an 11.8-fold increased thyroid cancer risk in HT patients after full adjustment for demographic and comorbidity confounders (21). Danis et al. similarly reported that HT was associated with elevated PTC risk and consequently recommended more aggressive diagnostic approaches for HT patients with thyroid nodules (22). These contradictory observations suggest HT’s dual role in thyroid carcinogenesis, potentially promoting initial malignant transformation while simultaneously constraining progression through immunomodulatory mechanisms.

Advancements in research methodologies have progressively elucidated the molecular mechanisms underlying HT’s influence on PTC pathogenesis. *In vitro* experiments by Kim et al. demonstrated that HT attenuates PTC cell invasiveness by upregulated of E-cadherin and TGF-β, key regulators of epithelial-mesenchymal transition (23). Complementarily, Sun et al.’s metabolomic profiling of PTC patients revealed dysregulated aminoacyl-tRNA biosynthesis and serine/threonine metabolism in HT-associated tumors, suggesting metabolic reprogramming as a contributory mechanism (24). Multi-omics analyses further identified HT-specific genetic signatures via The Cancer Genome Atlas, including DMBT1, MET, FAM20A, SERPINA1, CD53, FCER1G, and TYROBP (25–28). Most notably, Ma et al.’s single-cell RNA sequencing identified HT-associated cell clusters that establish a TSH-suppressive microenvironment through interactions among mTE3, nTE0, and nTE2 subpopulations. This immunomodulatory network is functionally mediated by the MIF-(CD74+CXCR4) signaling axis, facilitating stromal-immune communication that may concurrently promote carcinogenesis yet restrain progression (29).

Notably, our study identified preoperative misdiagnosis of contralateral PTC in 16.2% of presumed unilateral cases, with HT emerging as a negative correlate of bilateral involvement, a finding that diverged from the prevailing literature. This contrasts with Lv et al.’s analysis of 1442 patients undergoing total thyroidectomy for unilateral PTC with benign-appearing contralateral nodules, where HT was classified as a risk factor alongside multifocality and capsular invasion (30). Similarly, Wang et al.’s meta-analysis (23% contralateral malignancy misdiagnosis rate) implicated HT as a significant risk predictor (31). Conversely, Wu et al. reported no association between HT and contralateral cancer in their cohort (28.9% misdiagnosis rate) (32). Our large-scale propensity-matched cohort demonstrates HT’s negative correlation against contralateral malignancies, aligning with its established inhibitory roles in protection against CLNM in cN0 patients and LLNM in cN1b patients.

While this study demonstrates an inverse association between HT and markers of PTC aggressiveness, several limitations warrant consideration. Firstly, the retrospective nature inherently introduces selection bias. Secondly, although PSM mitigated major confounders, residual bias persists from unmeasured factors. Besides, the cohort’s restriction to established PTC cases precludes assessment of HT’s impact on *de novo* carcinogenesis risk, which requires prospective comparison between HT and non-HT populations. Further, our histopathology-based HT diagnosis, while the gold-standard, limits clinical practice where serological criteria (TPOAb/TgAb) or ultrasound features guide preoperative decisions. This methodological discordance may partially explain divergent findings in the literature regarding HT’s dual roles. Finally, while the results may suggest a potentially protective immune microenvironment in HT, the hypothesis remains unproven and requires validation by future molecular and immunopathological studies.

## Conclusion

This study demonstrates an inverse association between HT and markers of PTC aggressiveness, evidenced by reduced extrathyroidal extension, multifocality, and bilateral involvement alongside increased node-negative disease. After rigorous PSM, HT demonstrated inverse associations across key clinical scenarios: lowering CLNM rates in clinically node-negative patients, decreasing LLNM in cN1b subgroups, and reducing bilateral tumor detection among preoperatively diagnosed unilateral lesions.

## Data Availability

The raw data supporting the conclusions of this article will be made available by the authors, without undue reservation.
