# Supplementary material for: Hashimoto’s thyroiditis is associated with reduced invasiveness in papillary thyroid carcinoma: a propensity score-matched retrospective cohort study
Source: Front Immunol. 2026 Jan 21;16:1745452. doi: 10.3389/fimmu.2025.1745452 (PMC12867835; doi:10.3389/fimmu.2025.1745452)

Supplementary Material 1. Clinical characteristics of PTC patients after 1:3 propensity score matching.

| Characteristic | | Patients (N = 3753) | | | *P* value | OR (95%CI) |
| --- | --- | --- | --- | --- | --- | --- |
|  |  | Total  (n=3753) | HT absent (n = 2783) | HT present (n = 970) |  |  |
| Age ^a^, years | | 42 (34-50) | 42 (34-50) | 42 (33-51) | 0.737 | 0.999 (0.992-1.005) |
| Gender, n (%) | |  |  |  | 0.405 | 0.903 (0.710-1.148) |
|  | Female | 3347 (89.2) | 2475 (88.9) | 872 (89.9) |  |  |
|  | Male | 406 (10.8) | 308 (11.1) | 98 (10.1) |  |  |
| Clinical N stage, n (%) | |  |  |  | 0.187 |  |
|  | cN0 | 2859 (76.2) | 2141 (76.9) | 718 (74.0) |  |  |
|  | cN1a | 344 (9.2) | 247 (8.9) | 97 (10.0) | 0.215 | 1.171 (0.912-1.503) |
|  | cN1b | 550 (14.7) | 395 (14.2) | 155 (16.0) | 0.131 | 1.170 (0.954-1.435) |
| Clinical tumor location, n (%) | |  |  |  | 0.540 |  |
|  | Left side | 1413 (37.6) | 1036 (37.2) | 377 (38.9) |  |  |
|  | Right side | 1607 (42.8) | 1198 (43.0) | 409 (42.2) | 0.442 | 0.938 (0.797-1.104) |
|  | Isthmus | 4 (0.1) | 2 (0.1) | 2 (0.2) | 0.313 | 2.748 (0.386-19.578) |
|  | Bilateral | 729 (19.4) | 547 (19.7) | 182 (18.8) | 0.392 | 0.914 (0.745-1.122) |
| Surgical extent, n (%) | |  |  |  | 0.507 |  |
|  | Left side | 511 (13.6) | 379 (13.6) | 132 (13.6) |  |  |
|  | Right side | 509 (13.6) | 388 (13.9) | 121 (12.5) | 0.446 | 0.895 (0.674-1.190) |
|  | Bilateral | 2733 (72.8) | 2016 (72.4) | 717 (73.9) | 0.849 | 1.021 (0.823-1.267) |
| Tumor size ^a^, cm | | 0.8 (0.6-1.2) | 0.8 (0.6-1.2) | 0.8 (0.6-1.2) | 0.235 | 1.063 (0.961-1.176) |
| Tumor size, stratified, n (%) | |  |  |  | 0.863 |  |
|  | <0.5 cm | 908 (24.2) | 679 (24.4) | 229 (23.6) |  |  |
|  | 0.5-1 cm | 1648 (43.9) | 1228 (44.1) | 420 (43.3) | 0.883 | 1.014 (0.842-1.222) |
|  | 1-2 cm | 967 (25.8) | 710 (25.5) | 257 (26.5) | 0.503 | 1.073 (0.873-1.320) |
|  | 2-4 cm | 205 (5.5) | 149 (5.4) | 56 (5.8) | 0.535 | 1.114 (0.792-1.568) |
|  | >4 cm | 25 (0.7) | 17 (0.6) | 8 (0.8) | 0.444 | 1.395 (0.594-3.276) |
| Extrathyroidal extension | |  |  |  | 0.630 | 0.962 (0.820-1.128) |
|  | Absence | 1107 (29.5) | 815 (29.3) | 292 (30.1) |  |  |
|  | Presence | 2646 (70.5) | 1968 (70.7) | 678 (69.9) |  |  |
| Multifocal carcinoma | |  |  |  | 0.898 | 0.987 (0.851-1.146) |
|  | Absence | 1107 (29.5) | 1647 (59.2) | 577 (59.5) |  |  |
|  | Presence | 2646 (70.5) | 1136 (40.8) | 393 (40.5) |  |  |

^a^ Values are presented as median (Q1-Q3).

Abbreviations: PTC, papillary thyroid carcinoma; HT, Hashimoto's thyroiditis.

Supplementary Material 2. The methodology of propensity score matching about Hashimoto's thyroiditis.


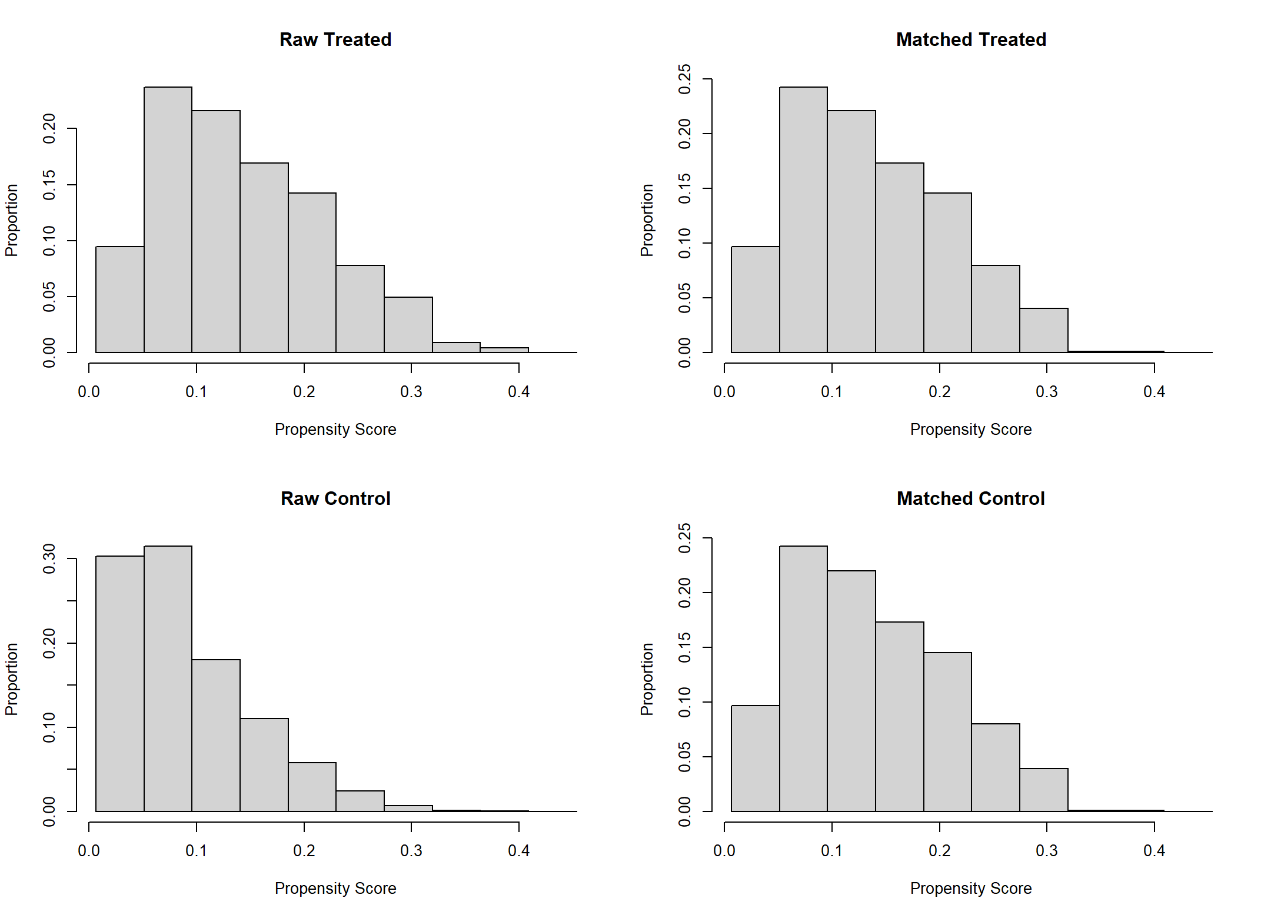


Supplementary Material 3. The distribution of propensity scores about Hashimoto's thyroiditis.


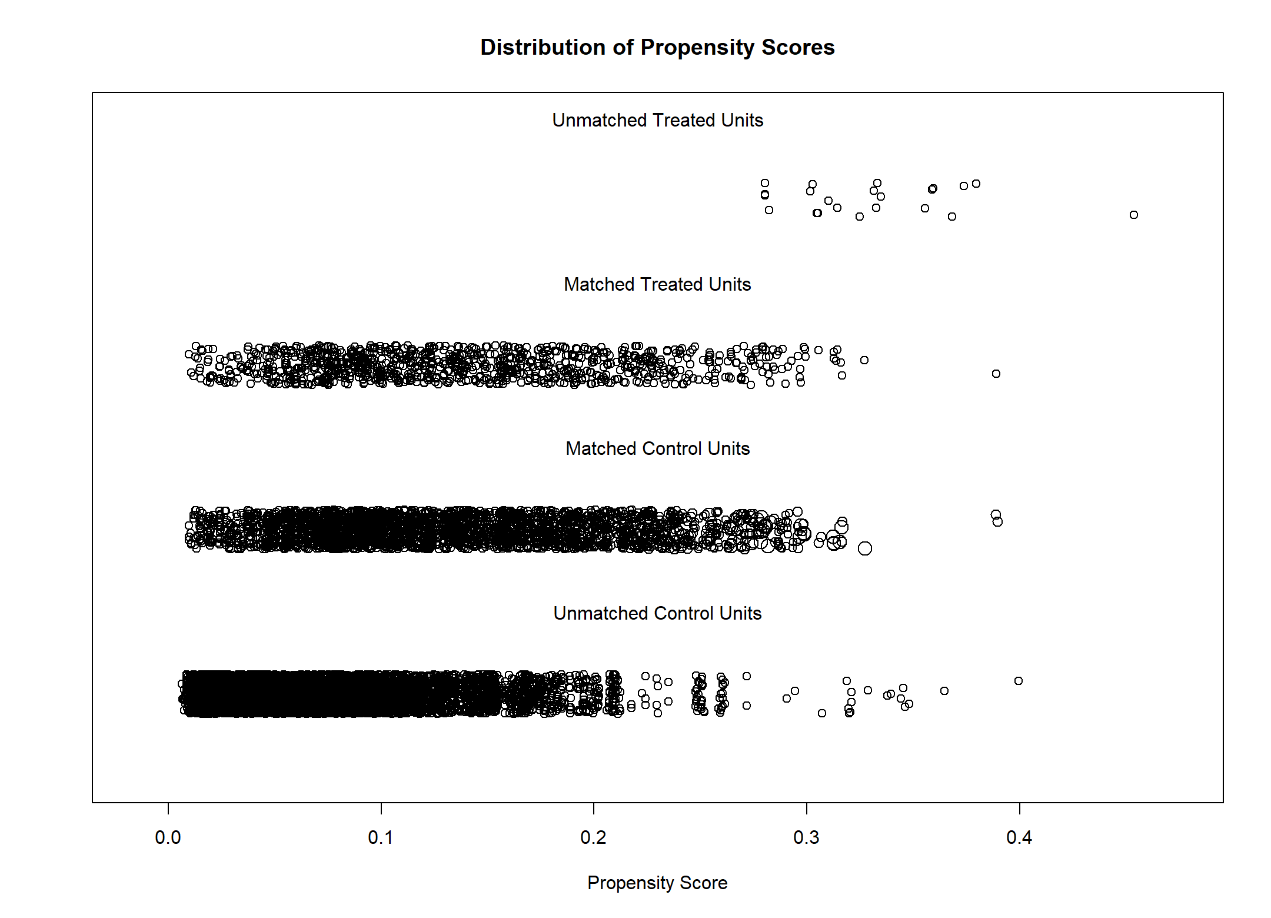

Supplement: Supplementary file 1 [file DataSheet1.docx]
